# BMAL1 facilitates trophoblast migration and invasion via SP1-DNMT1/DAB2IP pathway in recurrent spontaneous abortion

**DOI:** 10.18632/oncotarget.20702

**Published:** 2017-09-07

**Authors:** Shang Li, Junyu Zhai, Jiansheng Liu, Yan Hong, Weixiu Zhao, Aimin Zhao, Kang Sun, Yanzhi Du, Zi-Jiang Chen

**Affiliations:** ^1^ Center for Reproductive Medicine, Ren Ji Hospital, School of Medicine, Shanghai Jiao Tong University, Shanghai, China; ^2^ Shanghai Key Laboratory for Assisted Reproduction and Reproductive Genetics, Shanghai, China; ^3^ National Research Center for Assisted Reproductive Technology and Reproductive Genetics, The Key Laboratory for Reproductive Endocrinology of Ministry of Education, Shandong Provincial Key Laboratory of Reproductive Medicine, Center for Reproductive Medicine, Shandong Provincial Hospital, Shandong University, Jinan, China; ^4^ Department of Obstetrics and Gynecology, Ren Ji Hospital, School of Medicine, Shanghai Jiao Tong University, Shanghai, China

**Keywords:** BMAL1, recurrent spontaneous abortion, trophoblast, migration and invasion, progesterone, Pathology Section

## Abstract

The underlying mechanism about rhythms and epigenetics leading to aberrant trophoblast migration and invasion in recurrent spontaneous abortion (RSA) remains unknown. Brain and muscle ARNT-like protein 1 (BMAL1) is considered as a crucial role in fertility, and polymorphism of *BMAL1* gene has been reported to be associated with risk of miscarriage. However, the functional role of BMAL1 in RSA is not fully understood. Previous study shows the descended expression of DNA 5′-cytosine-methyltransferases 1 (DNMT1) in the villous of early pregnancy loss. Thus, understanding of the regulation of DNMT1 expression may be of significance for the elucidation of the process of RSA. Using HTR-8/SVneo and JEG-3 cell lines, we certified the induction of specificity protein 1 (SP1) to DNMT1 and DAB2 interaction protein (DAB2IP), respectively, both of which further activated matrix metallo-proteinase 2/9 (MMP2/9), bringing out changes in trophoblast migration and invasion. Notably, BMAL1 functioned as a positive upstream factor of SP1 only in HTR-8/SVneo cells but not in JEG-3 cells, inducing SP1-DNMT1/DAB2IP pathway and facilitating migration and invasion of trophoblasts. In addition, progesterone might restore the down-regulation of BMAL1 and downstream pathway in a dose-dependent manner. Last but not least, the decreased abundance of BMAL1 was correlated positively with that of SP1, DNMT1, DAB2IP, MMP2 and MMP9 in human villous specimens of RSA. Our results demonstrate that the induction of BMAL1 to SP1 contributes to the expression of DNMT1 and DAB2IP, respectively, activating trophoblast migration and invasion. The deregulation of the BMAL1-mediated pathway in RSA can be rescued by progesterone.

## INTRODUCTION

Recurrent spontaneous abortion (RSA), used to be defined as three or more spontaneous consecutive abortions prior to 20 weeks of gestation, impacts approximately 1 % - 2 % of couples. Now, some clinicians redefine RSA as two or more losses and this definition makes the percentage of RSA to 5 % [1, 2]. Abnormal embryonic karyotypes have been regarded as a major cause of RSA [3], such as trisomy, polyploidy or monosomy X arising de novo as a result of meiotic non-disjunction during gametogenesis. Besides, several etiological factors also account, such as parental chromosomal rearrangements, endocrine abnormalities, anatomical factors, immunologic factors, infectious diseases, inherited thrombophilic disorders, etc. [3]. However, at least 50 % of RSA patients have no deviations [4]. Molecular mechanisms still remain incompletely understood, especially with respect to the cases in which embryos are chromosomally normal.

The role of circadian rhythms in fertility has been paid more attention to in recent years. Female reproductive function is under strict circadian control by hypothalamic-pituitary-gonadal axis, and the suprachiasmatic nuclei (SCN) acts as the master cell-intrinsic circadian pacemaker [5]. The basic molecular circadian clock is composed of an auto-regulatory transcriptional feedback loop: a transcriptional activator complex formed by circadian locomotor output cycles kaput (CLOCK) and brain and muscle ARNT-like protein 1 (BMAL1) facilitates period 1/2 (PER1/2) and crytochrome 1/2 (CRY1/2), whose gene products then re-enter the nucleus and repress the transcription of the complex. Another loop promoted by CLOCK : BMAL1 complex and also suppressed by PER and CRY include REV-ERBα and RORα, both of which regulate the expression of BMAL1 by serving as the transcription repressor and activator, respectively [6–8].

As reported, the presence of circadian clock in female reproductive physiology participates in the generation of oestrus cycles, ovulation, implantation and the maintenance of pregnancy [9]. The high incidence of environmental disruption of circadian rhythms in our daily life is associated with an increase in the frequency of irregular menstrual cycles, alterations in serum LH and FSH levels, an increased risk of pre-term birth, and overall reduced fecundity [10–12]. *CLOCK* knock-down leads to reduction in reproduction and increased miscarriage risk in female mice [13], and *CLOCK* mutation reduces the implantation capacity of mice [14]. Previous studies also show that either *BMAL1* null mice or loss of *BMAL1* in ovarian steroidogenic cells in female mice results in implantation failure, and decreased *BMAL1* gene expression brings about down-regulation of *STAR* and thereby reduction of progesterone synthesis [15–17]. The reproductive capacity of women possessing polymorphism in *BMAL1* is assessed, and the number of miscarriages for this polymorphism is found greatly increased [18]. Thus, it is very likely that BMAL1 functions in RSA, while no direct evidence demonstrates its role in RSA and a limited amount of molecular and genetic data is drawn upon.

To further explore the downstream pathway of BMAL1, we focused on the following target genes: *DNA 5′-cytosine-methyltransferases 1 (DNMT1), DAB2 interaction protein (DAB2IP)* and *specificity protein 1 (SP1)*. Firstly, DNMT1 is one of major enzymes responsible for DNA methylation and indirectly involved in de novo methylation [19]. DNMT1 expression level is significantly down-regulated in the villous of early pregnancy loss (EPL), probably affecting embryonic implantation and development and giving rise to miscarriage [20]. Secondly, as a member of the Ras GTPase-activating family, DAB2IP has been implicated in the invasion in different types of cancers such as prostate, endometrial, breast, lung, hepatocellular and gastrointestinal cancers, etc. [21]. Recently, DAB2IP is reported strongly expressed in human villous but not in pre-eclampsia placentas [22], which implies its relationship with trophoblasts. Furthermore, previous studies demonstrate the role of SP1 as a transcription factor of *DNMT1* gene in several human cancer cells and mouse NIH3T3 cells [23–25]. *Sp1*^-/-^ embryos are retarded in development, show a broad range of abnormalities, and die around day 11 of gestation [26]. SP1 also positively participates in the invasion in various cancers [27]. In combination, we speculate the potential role of SP1 in miscarriage. To our knowledge, specific molecular mechanism associated with DNMT1, DAB2IP and SP1 in RSA has never been reported.

The current study aims to certify the crucial roles of BMAL1 and other downstream factors in promoting trophoblast migration and invasion in RSA, and to validate the underlying molecular mechanisms. Additionally, numerous clinical studies suggest a positive action of progesterone in the maintenance of pregnancy and prevention of miscarriage [28]. Here, we try to characterize the effect of progesterone on the associated pathway.

## RESULTS

### BMAL1 promoted migration and invasion in HTR-8/SVneo cells

**Figure 1:**
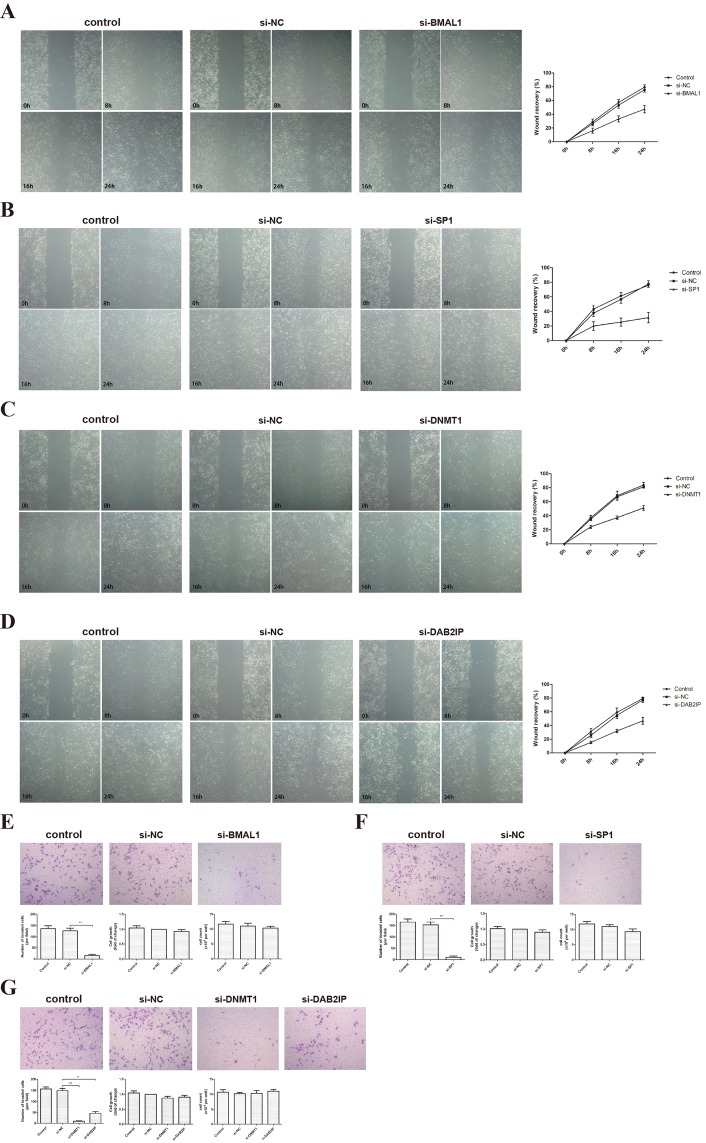
BMAL1, SP1, DNMT1 and DAB2IP induced migration and invasion of HTR-8/SVneo cells **A**. Scratch-wound assay after *BMAL1* knock-down (magnification: 50 ×). The left panel shows the representative images of Scratch-wound assay and the right panel clarifies the statistic results of the wound recovery of Scratch-wound assay. **B**. Scratch-wound assay after *SP1* knock-down (magnification: 50 ×). **C**. Scratch-wound assay after *DNMT1* knock-down (magnification: 50 ×). **D**. Scratch-wound assay after *DAB2IP* knock-down (magnification: 50 ×). **E**. Transwell assay and MTT after *BMAL1* knock-down (magnification: 100 ×). The top panel shows the representative images of transwell assay. The bottom panel from left to right presents the statistic result of invaded cells, the statistic result of MTT and the cell count. **F**. Transwell assay and MTT after *SP1* knock-down (magnification: 100 ×). **G**. Transwell assay and MTT after *DNMT1* or *DAB2IP* knock-down (magnification: 100 ×). Images are representative, and data are means ± SEM from three experiments. *P < 0.05, **P < 0.01 against si-NC cells.

HTR-8/SVneo, an immortalized human extravillous trophoblast (EVT) cell line, is widely used as a model for testing the invasion and migration of first-trimester EVTs. We examined the effect of BMAL1 on the migration of HTR-8/SVneo using a scratch-wound assay. Our results showed that BMAL1 induced the ability of HTR-8/SVneo cells to close the wound (Figure 1A). Similar changes were observed when we knocked down *SP1*, *DNMT1* or *DAB2IP* (Figure 1B, 1C, 1D). On the other hand, a transwell assay was used finding out that *BMAL1* siRNA treatment markedly decreased the invasion of HTR-8/SVneo cells compared with cells treated with negative control (NC) siRNA (Figure 1E). Moreover, the expression of BMAL1 impacted on the cell motility significantly, which was not caused by its effect on cell growth. Also, down-regulated *SP1, DNMT1* or *DAB2IP* weakened the invasion of HTR-8/SVneo cells (Figure 1F, 1G). Thus, these data revealed that BMAL1 potentially facilitated the migration and invasion of trophoblasts, as well as SP1, DNMT1 and DAB2IP.

### BMAL1 induced the expression of DNMT1 and DAB2IP via SP1 in HTR-8/SVneo cells, not in JEG-3 cells

**Figure 2:**
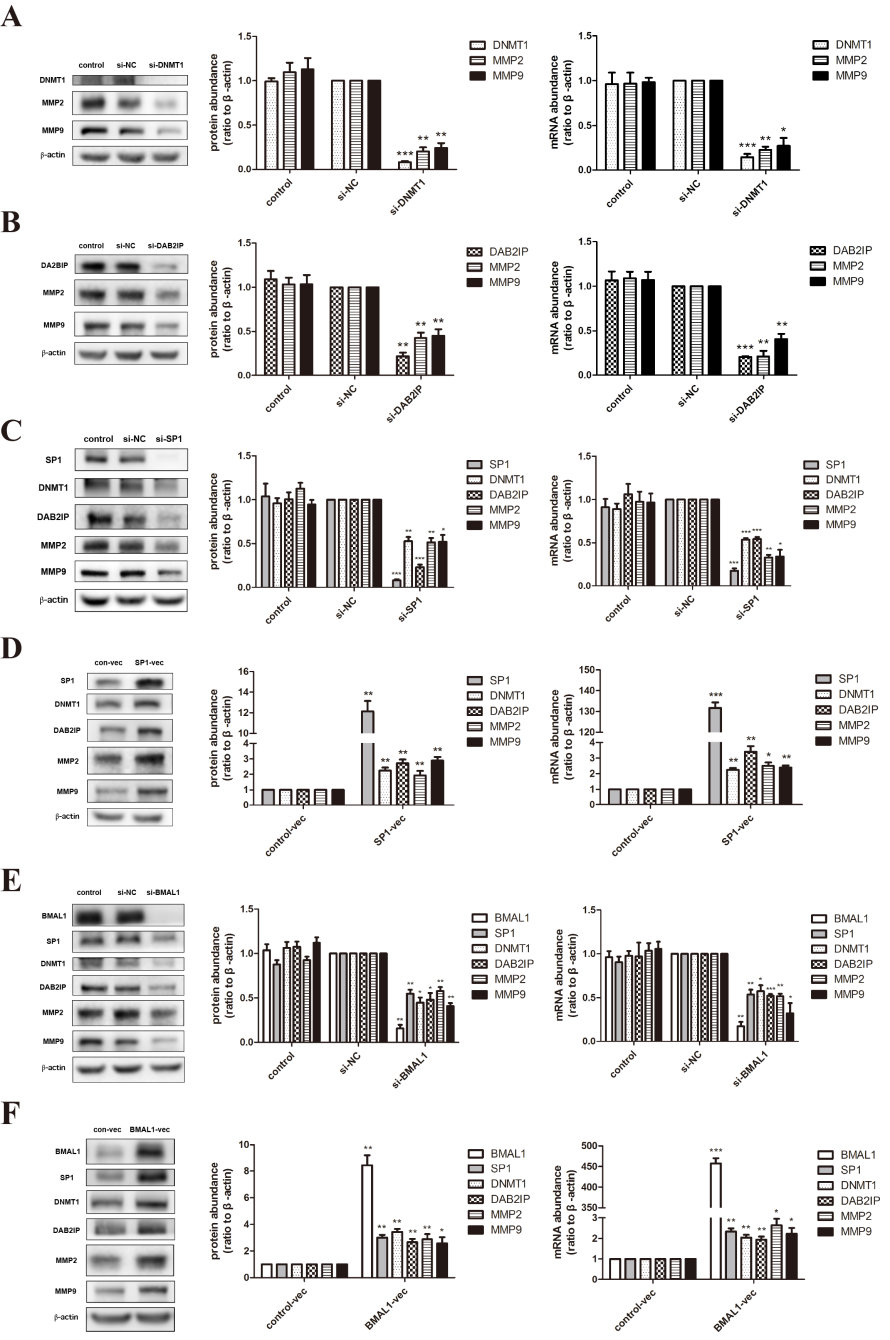
BMAL1 promoted SP1-induced DNMT1 and DAB2IP, and further regulated MMP2 and MMP9 in HTR-8/SVneo cells **A**. The mRNA and protein abundance of DNMT1, MMP2 and MMP9 after *DNMT1* knock-down in HTR-8/SVneo cells. The panel from left to right is the representative images of western blot assays, the immunoreactive bands densitometrically quantified and mRNA abundance. **B**. The mRNA and protein abundance of DAB2IP, MMP2 and MMP9 after *DAB2IP* knock-down in HTR-8/SVneo cells. **C**. The mRNA and protein abundance of SP1, DNMT1, DAB2IP, MMP2 and MMP9 after *SP1* knock-down in HTR-8/SVneo cells. **D**. The mRNA and protein abundance of SP1, DNMT1, DAB2IP, MMP2 and MMP9 after *SP1* over-expression in HTR-8/SVneo cells. **E**. The mRNA and protein abundance of BMAL1, SP1, DNMT1, DAB2IP, MMP2 and MMP9 after *BMAL1* knock-down in HTR-8/SVneo cells. **F**. The mRNA and protein abundance of BMAL1, SP1, DNMT1, DAB2IP, MMP2 and MMP9 after *BMAL1* over-expression in HTR-8/SVneo cells. β-Actin was used as a loading control. Blots are representative, and data are means ± SEM from four to five experiments. * *P* < 0.05, ** *P* < 0.01, *** *P* < 0.001 against si-NC cells or against control-vec cells.

**Figure 3:**
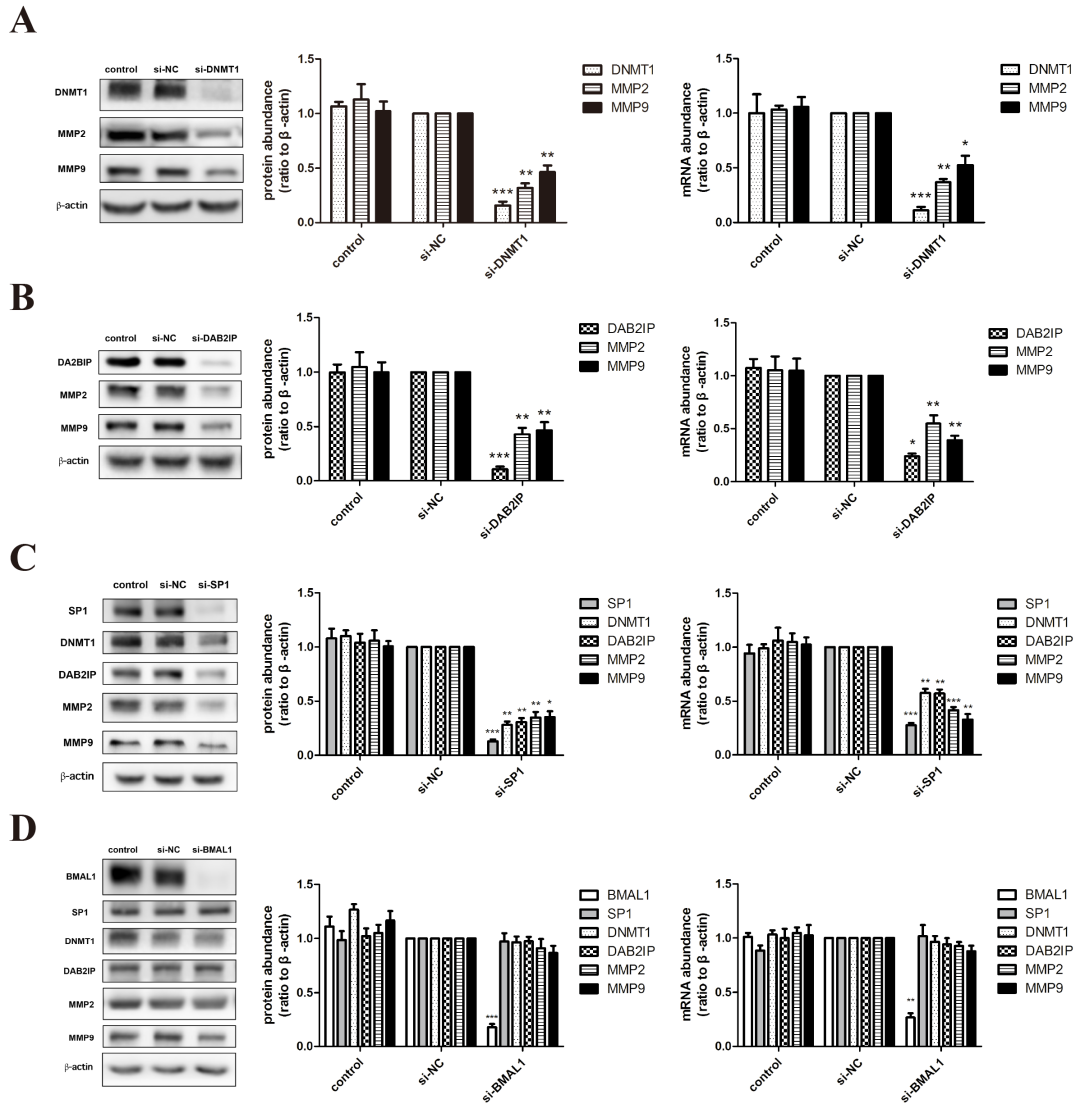
BMAL1 didn't function as an upstream factor of SP1-mediated DNMT1, DAB2IP and downstream pathway in JEG-3 cells **A**. The mRNA and protein abundance of DNMT1, MMP2 and MMP9 after *DNMT1* knock-down in JEG-3 cells. The panel from left to right is the representative images of western blot assays, the immunoreactive bands densitometrically quantified and mRNA abundance. **B**. The mRNA and protein abundance of DAB2IP, MMP2 and MMP9 after *DAB2IP* knock-down in JEG-3 cells. **C**. The mRNA and protein abundance of SP1, DNMT1, DAB2IP, MMP2 and MMP9 after *SP1* knock-down in JEG-3 cells. **D**. The mRNA and protein abundance of BMAL1, SP1, DNMT1, DAB2IP, MMP2 and MMP9 after *BMAL1* knock-down in JEG-3 cells. β-Actin was used as a loading control. Blots are representative, and data are means ± SEM from three to four experiments.* *P* < 0.05, ** *P* < 0.01,*** *P* < 0.001 against si-NC cells.

Based on the obvious phenomenon received above, we dissected the possible specific molecular mechanism of BMAL1 in trophoblast migration and invasion using HTR-8/SVneo (an immortalized human extravillous trophoblast cell line) and JEG-3 (a human choriocarcinoma cell line). After treatment of *DNMT1* siRNA or *DAB2IP* siRNA, we examined the mRNA and protein abundance of matrix metalloproteinase 2 (MMP2) and matrix metalloproteinase 9 (MMP9), known as key regulators and markers of trophoblast invasion [29]. The decreased expression tendency of these two MMPs (Figure 2A, 2B) was consistent with the decreased migration and invasion ability verified in HTR-8/SVneo cells. Likewise, same effects of DNMT1 and DAB2IP on MMP2 and MMP9 were observed in JEG-3 cells (Figure 3A, 3B).

Three SP1 putative binding sites are identified on the *DNMT1* promoter [30]. When we transfected *SP1* siRNA into HTR-8/SVneo cells, the mRNA and protein abundance of DNMT1, DAB2IP, MMP2 and MMP9 apparently decreased (Figure 2C). We also verified this regulatory relationship in JEG-3 cells (Figure 3C). On the contrast, over-expression of *SP1* using vector transfection led to the increase of DNMT1, DAB2IP and downstream MMP2 and MMP9 (Figure 2D). Hence, SP1 positively regulated the expression of DNMT1 and DAB2IP, respectively, in HTR-8/SVneo cells.

What's more, knock-down of *BMAL1* in HTR-8/SVneo cells brought out the reduction of SP1, DNMT1 and DAB2IP both in mRNA and protein levels, and further decreased MMP2 and MMP9 (Figure 2E). And the facilitation of BMAL1 to downstream factors after overexpressing *BMAL1* also verified this view (Figure 2F). However, there was no change tendency when knocking down *BMAL1* in JEG-3 cells (Figure 3D). Therefore, these data obviously indicated that BMAL1 was a critical upstream factor of SP1-mediated DNMT1 and DAB2IP activation and downstream responses of MMP2 and MMP9 in HTR-8/SVneo cells, while BMAL1 didn't act as an upstream factor in JEG-3 cells.

### Progesterone promoted BMAL1 and downstream factors in a dose-dependent manner

**Figure 4:**
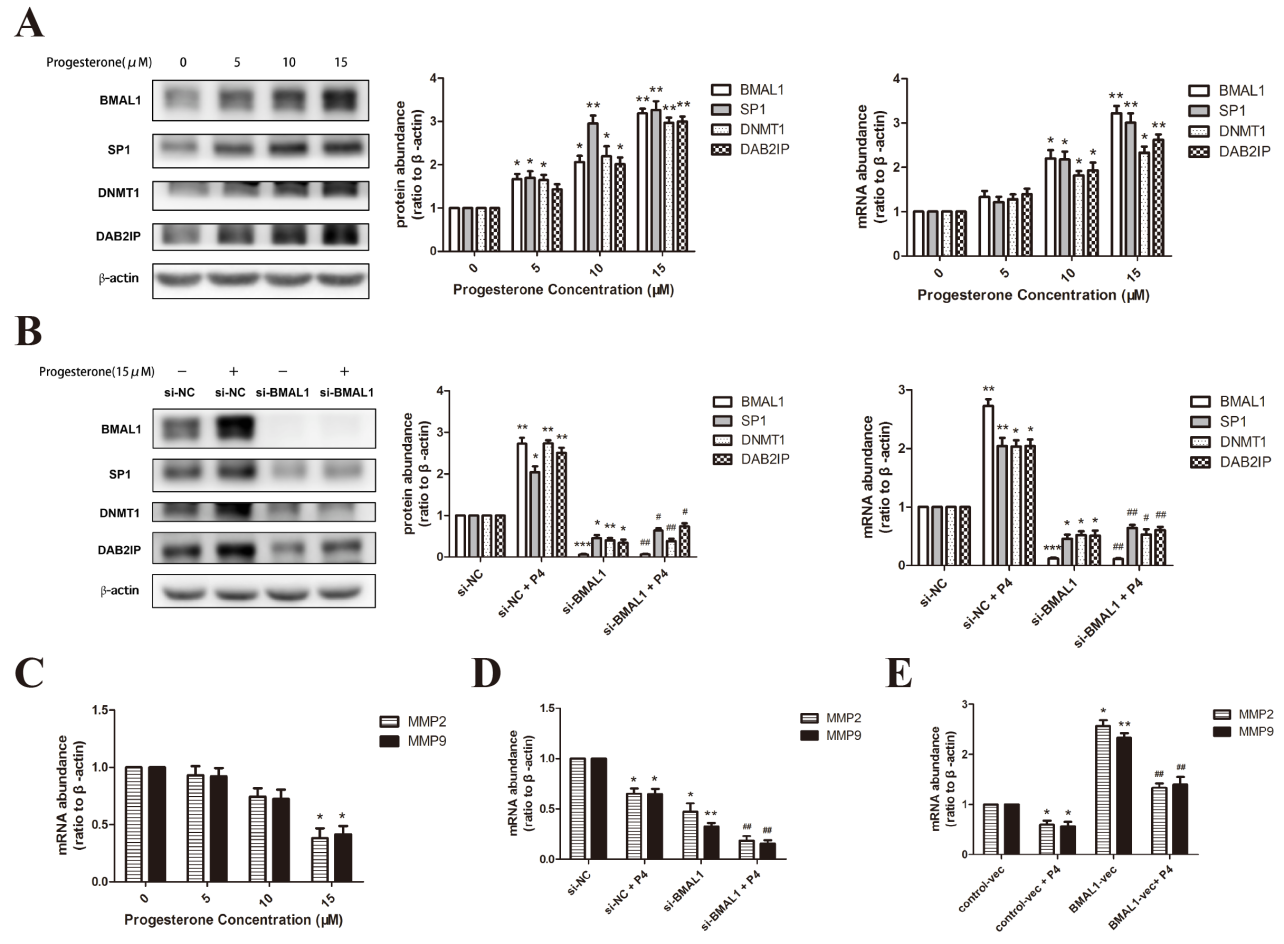
Progesterone promoted BMAL1-mediated pathway in HTR-8/SVneo cells **A**. The mRNA and protein abundance of BMAL1, SP1, DNMT1 and DAB2IP after treatment with progesterone for 24 hours at the concentration of 0, 5, 10 and 15 μmmol/L in HTR-8/SVneo cells. The panel from left to right is the representative images of western blot assays, the immunoreactive bands densitometrically quantified and mRNA abundance. **B**. The mRNA and protein abundance of BMAL1, SP1, DNMT1 and DAB2IP after *BMAL1* knock-down and further incubation with 15 μmmol/L progesterone for 24 hours in HTR-8/SVneo cells. **C**. The mRNA abundance of *MMP2* and *MMP9* after treatment with progesterone for 24 hours at the concentration of 0, 5, 10 and 15 μmmol/L in HTR-8/SVneo cells. **D**. The mRNA abundance of *MMP2* and *MMP9* after *BMAL1* knock-down and further incubation with 15 μmmol/L progesterone for 24 hours in HTR-8/SVneo cells. **E**. The mRNA abundance of *MMP2* and *MMP9* after *BMAL1* over-expression and further incubation with 15 μmmol/L progesterone for 24 hours in HTR-8/SVneo cells. β-Actin was used as a loading control. Blots are representative, and data are means ± SEM from three to four experiments. * *P* < 0.05, ** *P* < 0.01, *** *P* < 0.001 against si-NC cells or against control-vec cells; # *P* < 0.05, ## *P* < 0.01, ### *P* < 0.001 against si-NC+P4 cells or against control-vec +P4 cells.

Tissue concentrations of progesterone in human placenta are reported to vary from 1 to 10 μmmol/L [31]. So we treated HTR-8/SVneo cells with progesterone (Sigma Chemical Co., St. Louis, MO) at the concentration of 0, 5, 10 and 15 μmmol/L for 24 hours. The incubation caused the increase of BMAL1, SP1, DNMT1 and DAB2IP both in mRNA and protein levels, specifically in a dose-dependent manner (Figure 4A). Then we tried to elucidate the essential role of BMAL1 in the rescuing effect of progesterone on downstream pathway in RSA. After transfection of *BMAL1* siRNA for 24 hours, we further incubated HTR-8/SVneo cells with 15 μmmol/L progesterone for another 24 hours. As expected, the abundance of SP1, DNMT1 and DAB2IP in the group treated with transfection of *BMAL1* siRNA and progesterone apparently decreased compared with the group treated with non-specific scrambled siRNA and progesterone (Figure 4B).

However, progesterone had a negative effect on the expression of MMP2 and MMP9 at the concentration of 15 μmmol/L (Figure 4C), which was in accord with previous results [31]. And the treatment of *BMAL1* siRNA and progesterone (15 μmmol/L) further reduced *MMP2* and *MMP9* in mRNA level (Figure 4D). Then we treated HTR-8/SVneo cells with *BMAL1* vector for 48 hours and then progesterone (15 μmmol/L) for another 24 hours, finding out that the abundance of two MMPs obviously increased compared with the group treated with control vector and progesterone (15 μmmol/L) (Figure 4E). These data provided an evidence for the potential positive effect of progesterone on BMAL1-mediated promoting trophoblast migration and invasion, though there might be other molecular mechanisms underlying the regulating relationship between progesterone and MMP2/9.

### The expression of BMAL1 and its downstream factors SP1, DNMT1, DAB2IP, MMP2 and MMP9 in human villous specimens of RSA was decreased

**Figure 5:**
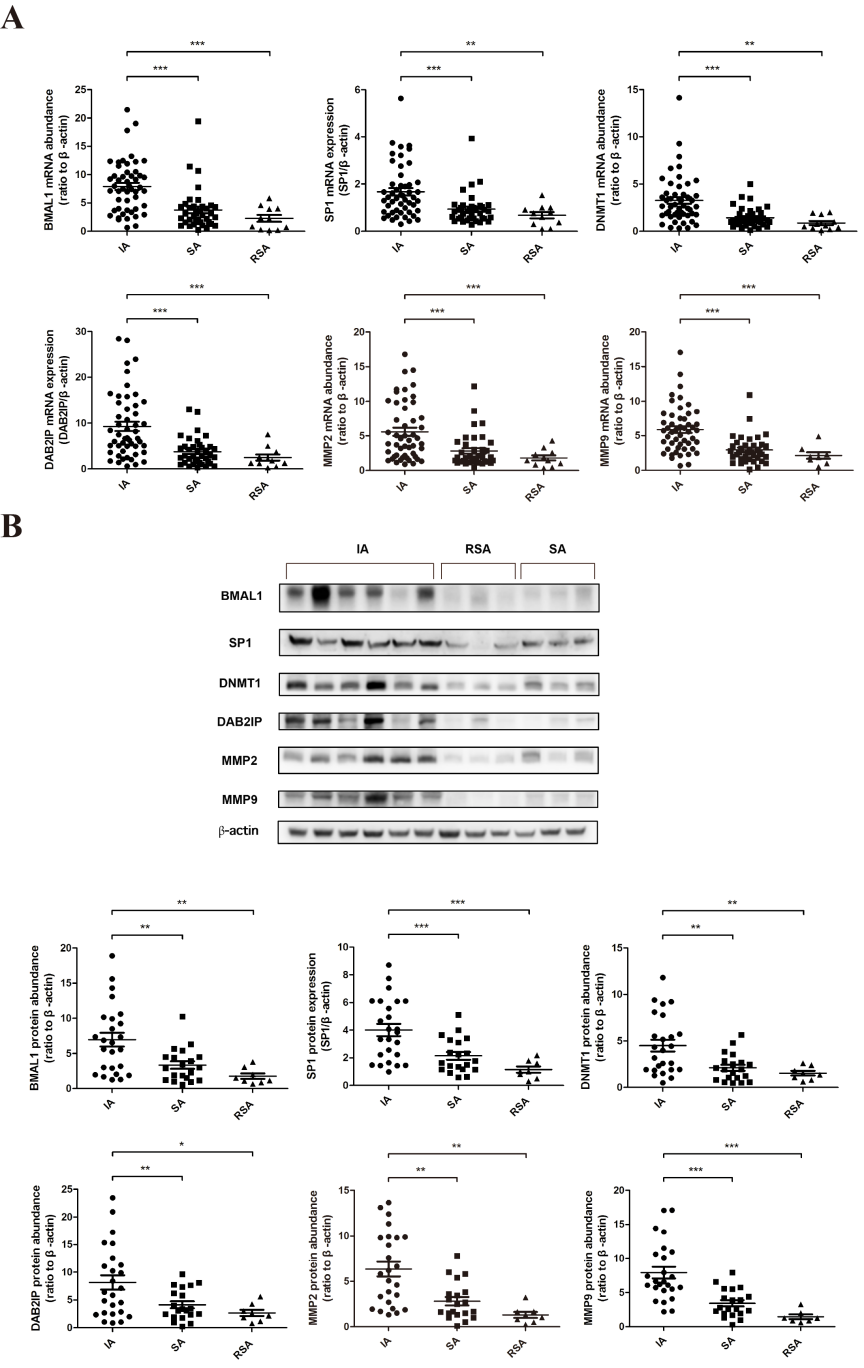
The decreased expression of BMAL1 in human villous specimens of RSA was consistent with SP1, DNMT1, DAB2IP, MMP2 and MMP9 **A**. The mRNA abundance of *BMAL1, SP1, DNMT1, DAB2IP, MMP2* and *MMP9* in human villous specimens. IA: induced abortion (*n* = 50). SA: sporadic abortion (*n* = 38). RSA: recurrent spontaneous abortion (n = 11). **B**. The protein abundance of BMAL1, SP1, DNMT1, DAB2IP, MMP2 and MMP9 in human villous specimens. The top panel shows the representative images of western blot assays. The bottom panel clarifies the proteins levels of detected specimens. IA: induced abortion (*n* = 25). SA: sporadic abortion (*n* = 20). RSA: recurrent spontaneous abortion (*n* = 8). β-Actin was used as a loading control. Blots are representative. * *P* < 0.05, ** *P* < 0.01, *** *P* < 0.001.

We examined the expression of BMAL1 and downstream factors using human villous specimens collected from sporadic abortion (SA) patients and RSA patients in comparison with villous from women undergoing induced abortion (IA) with nonmedical causes. The fetal chromosomal abnormality of RSA patients had been excluded. Decreased *BMAL1, SP1, DNMT1, DAB2IP, MMP2* and *MMP9* mRNA abundance was clearly detected in the villous of SA (*n* = 38) and RSA (*n*= 11) patients compared with that of women undergoing IA (*n* = 50) (Figure 5A). And the protein abundance of these genes was also reduced in the SA group (*n* = 20) and the RSA group (*n* = 8) compared with the IA group (*n* = 25) (Figure 5B). But there was no significant difference between the SA group and the RSA group both in mRNA and protein levels. Taken together, our human in vivo data was consistent with in vitro data from cell lines.

## DISCUSSION

Invasion of EVTs is fundamental to the development of placenta during early pregnancy, which is mediated by various signaling pathways and regulatory transcription factors. To form a functional placenta, fetal EVTs need to invade the maternal placental bed remodeling the maternal spiral arteries [32]. Inadequate trophoblast invasion can be found at the origin of various pregnancy complications and outcomes, such as pregnancy loss.

BMAL1, a circadian clock gene, is reported crucial for female reproduction especially during embryonic implantation [15, 17], and shares a potentially close relationship with miscarriage [18]. Daily variation in hypothalamic pituitary axis, including adrenocorticotropic hormone (ATCH) and cortisol, and chronotherapeutic success for preeclampsia both suggest the circadian regulation in spontaneous abortion [33]. In our study, we found that the abundance of BMAL1 was reduced in the villous specimens from RSA patients compared with that of women undergoing induced abortion and the loss of BMAL1 apparently decreased the migration and invasion of HTR-8/SVneo cells. Additionally, over-expression of *MMP2* and *MMP9* could rescue the decreased invasion ability of trophoblasts caused by *BMAL1* siRNA treatment in HTR-8/SVneo cells (Supplementary Figure. 1), which suggested that BMAL1 might induce the invasion of trophoblasts via MMP2 and MMP9. These in vivo and in vitro data firstly presented the direct evidence implicating the essential role of BMAL1 in RSA.

Epigenetic changes have been brought out by histone modifications, histone variants, DNA methylation, small RNAs and/or a combination of all these events. Aberrant DNA methylation gives rise to serious human diseases [19]. Arising during embryo development or gametogenesis, it's also been suggested as a potential cause of pregnancy loss. Extreme DNA methylation values at several imprinted loci were more frequent in the muscle tissue of spontaneous abortions and still births [34]. Also, more outliers in DNA methylation at seven imprinted loci were observed in the villous of RSA [35]. What's more, 5-aza-dC (an inhibitor of DNA methylation) treatment significantly induced mesenchymal-to-epithelial transition in HTR8/SVneo cells [36]. We detected the abundance of DNMT1 in the villous of RSA, the reduced change trend of which kept consistent with the data previously reported in EPL. And knock-down of *DNMT1* resulted in decreased trophoblasts migration and invasion, as well as decreased expression of MMP2 and MMP9 both in HTR-8/SVneo cells and JEG-3 cells. DNMT1 has been previously regarded essential in the regulation of differentiation of extra-villous trophoblasts [37]. Here, we certified its positive role in the migration and invasion of trophoblasts. However, we didn't detect its methylation of MMP2/9 in this pathway because they were positively modulated by DNMT1 while the methylation function is to silence the target gene. The specific mechanism between DNMT1 and MMP2/9 remains to be further researched.

Abnormal expression of DAB2IP is reported related to excessive oxidative stress in pre-eclampsia [22]. DAB2IP was observed lowly expressed in the villous of RSA. After silencing DAB2IP, we detected the migration and invasion ability of HTR-8/SVneo cells and the expression of *MMP2* and *MMP9* in HTR-8/SVneo cells and JEG-3 cells. We got the same phenomenon just as silencing DNMT1. Accordingly, we assumed that loss of DAB2IP in RSA repressed MMP2 and MMP9, inhibiting the migration and invasion of trophoblasts. Besides the previously reported involvement in human cancer cells, DAB2IP shows a potentially imperative role in trophoblasts and pathologic pregnancy. The specific regulation of DAB2IP to MMP2/9 needs to be carefully studied in the future.

From HTR-8/SVneo and JEG-3 cells tested, we showed that SP1 facilitated the expression of DNMT1, which is consistent with previous reports in various human cancers and mouse NIH3T3 cells [23–25]. We also certified the positive role of SP1 in regulating DAB2IP and further promoting MMP2 and MMP9. In addition, the sharply reduced expression of SP1 in the villous of RSA was in accord with the declined migration and invasion of HTR-8/SVneo cells after silencing *SP1*. To sum up, SP1 increased the expression of DNMT1 and DAB2IP, respectively, both of which further induced MMP2 and MMP9, thereby contributing to the migration and invasion of trophoblasts. SP1 has been regarded as one of vital regulators in vertebrate embryogenesis [38], as well as in male germ cell development and differentiation [39]. Our findings further verified the crucial role of SP1 in the embryonic development.

The relationship between circadian rhythms and DNA methylation has been arousing an extensive concern. While numerous researchers pay more attention to the methylation of promoters of rhythmic genes, we focused on the specific molecular mechanism revealing the potent role of DNMT1. Apart from the two classic feedback loops, BMAL1 also affects thousands of other downstream target genes known as clock-controlled genes involved in rhythmic biological processes [7]. We illustrated that BMAL1 induced the expression of SP1 and further facilitated the downstream pathway. However, the regulation of BMAL1 to SP1 occurred only in HTR-8/SVneo cells but not in JEG-3 cells. JEG-3 is a kind of human choriocarcinoma cell line in which our target genes are expressed strongly and stably. HTR-8/SVneo is an immortalized human extravillous trophoblast cell line whose physiological environment is relatively “similar” to human body compared to cancerous JEG-3 cells. Further investigations need to be done to elaborate this phenomenon.

As we all known, progesterone is essential for the maintenance of pregnancy. During the time 5 - 10 days after the LH surge, the endometrium is the most conducive to blastocyst implantation and subsequent development, which won't take place if the secretion of progesterone is below physiological value [40]. Animal models also show that high local concentration of progesterone facilitates implantation of xenogenic cultured cells in the uterus [41]. As regards trophoblast invasion, 20 μmmol/L progesterone was reported to significantly inhibit both invasion capacity and MMP2/9 activity in HTR-8/SVneo cells [31]. It was also reported that progesterone inhibited leptin-induced invasiveness of BeWo cells, another human trophoblastic cell line [42]. Likely, we observed the inhibition of progesterone (15 μmmol/L) on the expression of MMP2/9 in HTR-8/SVneo cells. These effects could be illustrated through the classical progesterone response elements on *MMP2/9* promoters and the progesterone - progesterone receptor (PR) complex might also interact with other proteins participating in the regulation [43]. However, we delineated the positive role of progesterone in BMAL1-mediated promoting trophoblast migration and invasion by inducing the BMAL1-mediated SP1-DNMT1/DAB2IP pathway in a dose-dependent manner. Note that this promotion of progesterone would be ineffective in absent of BMAL1. Taken together, though having an eventually stronger effect on inhibiting trophoblast migration and invasion, progesterone could actually indeed rescue the down-regulation of BMAL1 and downstream pathway in RSA. Since BMAL1 has been always identified as facilitation to progesterone synthesis [15], our results could be considered as a positive feedback of it. In the murine uterus, BMAL1 contains putative PR-binding locations and displays up-regulated expression after progesterone administration in vivo [44]. So we speculated that progesterone might interact with PR and then the complex bound with BMAL1 regulating its expression. However, it's unknown whether the relationship between BMAL1 and PR is conserved between rodents and humans. Thus, to elucidate the complete role of progesterone on BMAL1 and trophoblast functions, further research is required to be done.

**Figure 6:**
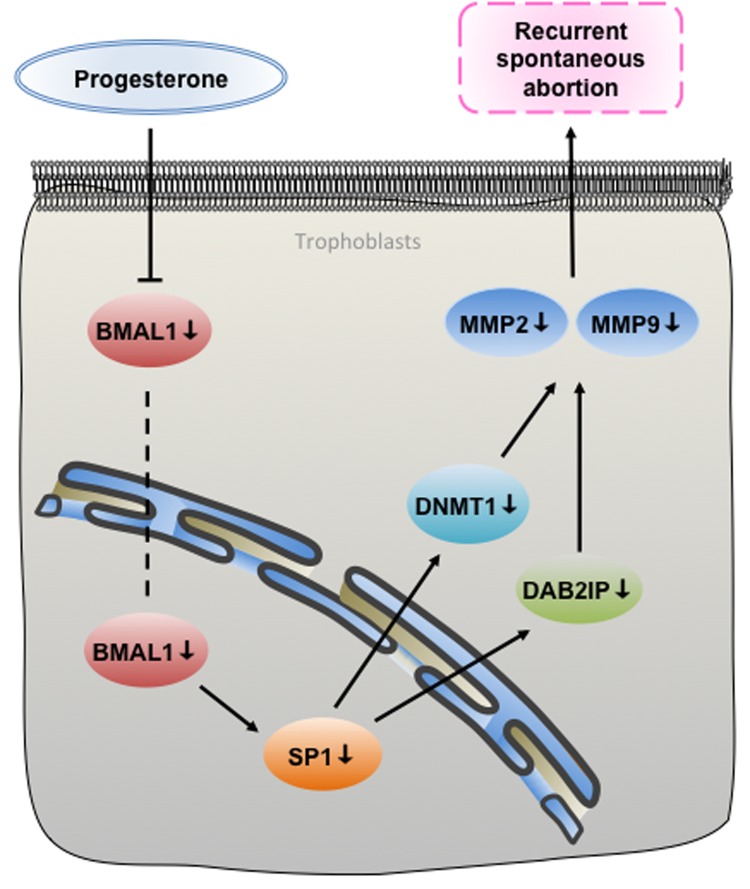
A proposed signaling pathways underpinning that BMAL1 induced trophoblast migration and invasion in recurrent spontaneous abortion As an upstream factor of SP1, BMAL1 facilitated DNMT1 and DAB2IP, respectively, and then promoted migration and invasion of trophoblasts via MMP2 and MMP9. Additionally, the deregulation of the BMAL1-mediated pathway in RSA can be rescued by progesterone.

In summary, our study reveals that BMAL1 functioned as a positive upstream factor of SP1 contributing to the expression of DNMT1 and DAB2IP, respectively, promoting trophoblast migration and invasion via MMP2 and MMP9. And the deregulation of the BMAL1-mediated pathway in RSA can be rescued by progesterone in a dose-dependent manner (Figure 6). We demonstrate the functional role of BMAL1 in RSA, especially in cases excluding fetal chromosomal abnormality, which explains how loss of BMAL1 underlies the onset of descended migration and invasion of trophoblasts. The above results prompt that subtle environmental perturbations during embryonic development could potentially disrupt the setting of epigenetic markers and eventually give rise to RSA. Once more, we present another new molecular mechanism for the usage of progesterone in preventing spontaneous abortion, concerning about rhythms. We are convinced that the assessment of BMAL1 and other downstream factors expression in RSA specimens can be a valuable therapeutic target.

## MATERIALS AND METHODS

### Clinical specimens

Appropriate informed consents were obtained from all patients and the tissue procurement protocol in this study was approved by the Institutional Review Board of Ren Ji Hospital, School of Medicine, Shanghai Jiao Tong University (approval number 2015071708). Villous specimens were collected from SA patients, RSA patients (three or more times abortion) and women undergoing IA with nonmedical causes. All operations were performed between 9:00 - 11:00 am. Cases with parental chromosomal, autoimmune, hormonal, anatomic and infectious causes, and complications such as hypertension, diabetes, thyroid abnormalities, etc. were excluded from this study. Notably, we excluded fetal chromosomal abnormality in collected villous specimens of RSA patients. The clinical information of patients is presented in Supplementary Table 1.

### Array CGH analysis

Part of tissue, separated from villous specimen collected from RSA patients, was sent to Shandong Provincial Key Laboratory of Reproductive Medicine, Center for Reproductive Medicine, Shandong Provincial Hospital, Shandong University to conduct array CGH analysis. Only specimens identified no chromosomal abnormalities by Array CGH were selected for further investigations.

### Cell culture

HTR-8/SVneo (an immortalized human extravillous trophoblast cell line) and JEG-3 (a human choriocarcinoma cell line) were kindly provided by Fudan University. They were cultured in Phenol Red-free DMEM/F-12 medium (Gibco, Grand Island, NY) containing 10 % fetal bovine serum (FBS) (Gibco) and incubated at 37 °C in a humidified atmosphere with 5 % CO_2_. The cell lines were routinely subcultured every 3 days. Cells used for experiments were controlled at passage 5 – 10.

### Transfection of small interfering (si) RNA with liposome

Transfections of siRNA were performed using RNAiMAX (Invitrogen, Carlsbad, CA, USA) according to the manufacturer's instrument. We refreshed the medium after culturing HTR-8/SVneo cells in the six-well tissue culture plate (Sigma Chemical Co., St. Louis, MO) for 24 hours. The mixture of RNAiMAX and 50 pmol siRNA (GenePharma Co., Shanghai, China) against *BMAL1* (5’- CCACCAACCCAUACACAGAAGCAAA-3’), or *SP1* (5’- CCAGCAACAUGGGAAUUAUTT-3’), or *DNMT1* (5’- GCCUCAUCGAGAAGAAUAUTT-3’), or *DAB2IP* (5’- GGAGCGCAACAGUUACCUG-3’), or non-specific scrambled siRNA (5’- UUCUCCGAACGUGUCACGUTT-3’) as negative control in Opti-MEM (Gibco) was evenly added into each well. Then the cells were further incubated for 48 hours before the next treatment. The efficiency of knock-down was assessed by measuring mRNA and protein abundance of target genes.

### Transfection of plasmids with electroporation

HTR-8/SVneo cells (2 × 10^6^) were mixed with 5 μg/well pENTER-BMAL1, p3*flag-cmv-10-SP1, pCMV3-flag-His-DNMT1, pCMV-C-flag-MMP2 or pCMV-C-flag-MMP9 (Transheep, Shanghai, China), respectively, in OPTI-MEM (Gibco). Then the mixture was added into 2-mm gap cuvettes and electroporated at 155V for 5 ms using a NEPA21 electroporator (Nepa Gene, Chiba, Japan). After dilution with DMEM/F-12 medium containing 10 % FBS, the cells were transferred into a six-well plate and were ready for the treatment after further incubation for 72 hours. The efficiency of over-expression was assessed by measuring mRNA and protein abundance of target genes.

### Scratch-wound assay

After transfection of siRNA of *BMAL1, SP1, DNMT1, DAB2IP* or randomly scrambled siRNA, respectively, with HTR-8/SVneo cells for 48 hours, a denuded area was created across the diameter of well by a blue tip. Then the cells were washed with PBS and incubated in DMEM/F-12 medium containing 1 % FBS. Phase-contrast images were taken at 50 x magnification using a microscope (Zeiss, Oberkochen, Germany) at a time point of 0, 8, 16 and 24 hours of incubation. The percentage of areas covered by migrated cells (wound recovery) was calculated as an indication of the migration ability.

### Transwell assay

The 24-well plate equipped with 8-mm pore size of polycarbonate membrane transwell inserts (Corning, New York, USA) was used to carry out the transwell assay. Each insert was carefully added with 100 μl of the diluted Corning Matrigel matrix coating solution and dried at 37 °C for one hour prior to conducting the assay. After allowing 48 hours for the transfection of siRNA of *BMAL1, SP1, DNMT1, DAB2IP* and negative control (NC), respectively, HTR-8/SVneo cells were refreshed with DMEM/F-12 medium containing 1 % FBS. 6 hours later, 5 × 10^4^ cells/insert were seeded and incubated in 100 μl DMEM/F-12 medium containing 1 % FBS. All inserts were placed into the 24-well plates containing 600 μl/well of DMEM/F-12 medium with 20 % FBS. After incubation for 48 hours, the non-invaded cells on the upper surface of the membrane were gently removed by a cotton swab. The invaded cells on the under surface were first fixed in 90 % ethanol for 30 min, then stained with 0.1 % crystal violet for 20 min, and finally washed three times with phosphate buffer saline (PBS). Five randomly selected fields of cells were photographed at 100 x magnification and quantified. The number of invaded cells of each field was counted as an indication of the invasion ability.

### MTT assay

The 3- (4, 5-dimethylthiazol-2-yl)-2,5-diphenyltetrazolium bromide-MTT-assay was used for evaluating the effect of transfection of siRNA on cell viability of HTR-8/SVneo cells. After allowing 48 hours for the transfection, HTR-8/SVneo cells were suspended and seeded into a 24-well plate at a density of 5 × 10^4^ cells/well in 500 μl DMEM/F-12 medium containing 10 % FBS. After 48 hours, as same as the incubation of transwell assay, 50 μl MTT (5 mg/mL) was added into each well and the plates were incubated for further 4 hours at 37 °C. Then the medium was removed and 500 μl dimethylsulfoxide (DMSO) was added into each well and the plates were shaken for 20 min at room temperature. Finally, the optical density (OD) values was measured at 570 nm using Infinite M200 Pro (Tecan, Männedorf, Switzerland), which were regarded as the cell viability.

### Quantitative real-time polymerase chain reaction (qRT-PCR)

Total RNA was extracted from the above treated cells and villous specimens using a total RNA kit following a protocol provided by the manufacturer (FOREGENE, Chengdu, China). RNA concentration and quality were determined by measuring OD260 (optical density at 260 nm) and the ratio of OD260/OD280 with NanoDrop ND-2000. mRNA from the total cellular RNA was reverse-transcribed to cDNA using PrimeScript RT Master Mix Perfect Real Time kit (TaKaRa, Dalian, China). The absolute mRNA in each sample was calculated according to a standard curve set up using serial dilutions of known amounts of specific templates against corresponding cycle threshold (Ct) values. The housekeeping gene *β-actin* was amplified in parallel as an internal loading control. The primer sequences used of targeting genes were as follows: *BMAL1*, 5’-TGCAAGGGAAGCTCACAGTC-3’ (forward) and 5’ -GATTGGTGGCACCTCTTAATG-3’ (reverse); *SP1*, 5’ -TGCCTCCACTTCCTCGATTT-3’ (forward) and 5’ -TCTGGTGGGCAGTATGTTGT-3’ (reverse); *DNMT1*, 5’ -AGAACGGTGCTCATGCTTACA-3’ (forward) and 5’ -CTCTACGGGCTTCACTTCTTG-3’ (reverse); *DAB2IP*, 5’ -CTGAGCGGGATAAGTGGATGG-3’ (forward) and 5’ -AAACATTGTCCGTCTTGAGCTT-3’ (reverse); *MMP2*, 5’ -GATACCCCTTTGACGGTAAGGA-3’ (forward) and 5’ -CCTTCTCCCAAGGTCCATAGC-3’ (reverse); *MMP9*, 5’ -AGACCTGGGCAGATTCCAAAC-3’ (forward) and 5’ -CGGCAAGTCTTCCGAGTAGT-3’ (reverse); *β-actin*, 5’ -GGGAAATCGTGCGTGACATTAAG-3’ (forward) and 5’ -TGTGTTGGCGTACAGGTCTTTG-3’ (reverse). The ratio of the target gene over *β-actin* was obtained as the target mRNA levels.

### Western blotting

Total cellular protein was extracted from the above treated cells and villous specimens using ice-cold radioimmunoprecipitation assay (RIPPA) lysis buffer (Cwbiotech, Beijing, China) containing a protease inhibitor cocktail (Roche, Basel, Switzerland). The abundance of BMAL1, SP1, DNMT1 and DAB2IP was determined using a standard Western blotting protocol. Briefly, after determination of protein concentration using a Pierce BCA Protein Assay Kit (Thermo Scientific), 30 mg protein from each sample was electrophoresed in 8 % SDS–polyacrylamide gel and transferred to the nitrocellulose membrane (Millipore, Billerica, MA). After blocking with 5 % nonfat milk, the membrane was incubated with BMAL1 antibody (1:500; Abcam, Cambridge, UK), SP1 antibody (1:500; Santa Cruz Biotechnology, Santa Cruz, CA), DNMT1 antibody (1:1000; Abcam), DAB2IP antibody (1:2000; Abcam), MMP2 antibody (1:500; Proteintech, Wuhan, China), MMP9 antibody (1:500; Proteintech) respectively, overnight at 4 °C. After incubation with the appropriate secondary antibody conjugated with horseradish peroxidase (Proteintech) for 1.5 h at room temperature, the enhanced chemiluminescent detection system (Millipore, Billerica, MA) was used to detect the bands with peroxidase activity. The bands were visualized using a G-Box iChemi Chemiluminescence image capture system (Syngene, Haryana, India). To control sampling error, the same blot was also probed for β-actin (1:3000; BD, Billerica, USA) as an internal loading control. The ratio of band intensities of target protein over β-actin was obtained as the target protein levels.

### Statistical analysis

All data are represented as mean ± SEM. Each experiment was repeated several times (n = 3 - 5). After examination for normal distribution, data analysis was performed using paired Student's t test or one-way ANOVA test followed by the Newman-Keuls multiple comparison test was used where appropriate to assess significant differences. Significance was set at *p* < 0.05.

## SUPPLEMENTARY MATERIALS FIGURES AND TABLES



## Abbreviations

BMAL1: brain and muscle ARNT-like protein 1
SP1: specificity protein 1
DNMT1: DNA 5′-cytosine-methyltransferases 1
DAB2IP: DAB2 interaction protein
MMP2/9: matrix metallo-proteinase 2/9
SCN: suprachiasmatic nuclei
CLOCK: circadian locomotor output cycles kaput
PER1/2: period 1/2
Cry 1/2: Cryptochrome1/2
SIRT1: silent mating type information regulation 2 homolog 1
EPL: early pregnancy loss
RSA: recurrent spontaneous abortion
SA: sporadic abortion
IA: induced abortion
EVT: extravillous trophoblast
siRNA: small interfering RNA

## References

[1] Practice Committee of American Society for Reproductive Medicine (2013). Definitions of infertility and recurrent pregnancy loss: a committee opinion. Fertil Steril.

[2] Ford HB, Schust DJ (2009). Recurrent pregnancy loss: etiology, diagnosis, and therapy. Rev Obstet Gynecol.

[3] Garrido-Gimenez C, Alijotas-Reig J (2015). Recurrent miscarriage: causes, evaluation and management. Postgrad Med J.

[4] Rai R, Regan L (2006). Recurrent miscarriage. The Lancet.

[5] Miller BH, Takahashi JS (2013). Central circadian control of female reproductive function. Front Endocrinol (Lausanne).

[6] Lowrey PL, Takahashi JS (2011). Genetics of circadian rhythms in Mammalian model organisms. Adv Genet.

[7] Mohawk JA, Green CB, Takahashi JS (2012). Central and peripheral circadian clocks in mammals. Annu Rev Neurosci.

[8] Takahashi JS (2015). Molecular components of the circadian clock in mammals. Diabetes Obes Metab.

[9] Kennaway DJ, Boden MJ, Varcoe TJ (2012). Circadian rhythms and fertility. Mol Cell Endocrinol.

[10] Baker FC, Driver HS (2007). Circadian rhythms, sleep, and the menstrual cycle. Sleep Med.

[11] Lawson CC, Whelan EA, Lividoti Hibert EN, Spiegelman D, Schernhammer ES, Rich-Edwards JW (2011). Rotating shift work and menstrual cycle characteristics. Epidemiology.

[12] Amaral FG, Castrucci AM, Cipolla-Neto J, Poletini MO, Mendez N, Richter HG, Sellix MT (2014). Environmental control of biological rhythms: effects on development, fertility and metabolism. J Neuroendocrinol.

[13] Li R, Cheng S, Wang Z (2015). Circadian clock gene plays a key role on ovarian cycle and spontaneous abortion. Cell Physiol Biochem.

[14] Amano T, Anzai M, Matsumoto K (2016). The Clock mutation reduces reproductive performance of mice by affecting the implantation capacity: Maternal Clock mutation is not the only factor affecting implantation. Theriogenology.

[15] Liu Y, Johnson BP, Shen AL, Wallisser JA, Krentz KJ, Moran SM, Sullivan R, Glover E, Parlow AF, Drinkwater NR, Schuler LA, Bradfield CA (2014). Loss of BMAL1 in ovarian steroidogenic cells results in implantation failure in female mice. Proc Natl Acad Sci U S A.

[16] Boden MJ, Varcoe TJ, Voultsios A, Kennaway DJ (2010). Reproductive biology of female Bmal1 null mice. Reproduction.

[17] Xu J, Li Y, Wang Y, Xu Y, Zhou C (2016). Loss of Bmal1 decreases oocyte fertilization, early embryo development and implantation potential in female mice. Zygote.

[18] Kovanen L, Saarikoski ST, Aromaa A, Lönnqvist J, Partonen T (2010). ARNTL (BMAL1) and NPAS2 gene variants contribute to fertility and seasonality. PLoS One.

[19] Dean W, Lucifero D, Santos F DNA methylation in mammalian development and disease. Birth Defects Res C Embryo Today.

[20] Yin LJ, Zhang Y, Lv PP, He WH, Wu YT, Liu AX, Ding GL, Dong MY, Qu F, Xu CM, Zhu XM, Huang HF (2012). Insufficient maintenance DNA methylation is associated with abnormal embryonic development. BMC Med.

[21] Maertens O, Cichowski K (2014). An expanding role for RAS GTPase activating proteins (RAS GAPs) in cancer. Adv Biol Regul.

[22] Shan N, Xiao X, Chen Y, Luo X, Yin N, Deng Q, Qi H (2016). Expression of DAB2IP in human trophoblast and its role in trophoblast invasion. J Matern Fetal Neonatal Med.

[23] Liu S, Liu Z, Xie Z, Pang J, Yu J, Lehmann E, Huynh L, Vukosavljevic T, Takeki M, Klisovic RB, Baiocchi RA, Blum W, Porcu P (2008). Bortezomib induces DNA hypomethylation and silenced gene transcription by interfering with Sp1/NF-kappaB-dependent DNA methyltransferase activity in acute myeloid leukemia. Blood.

[24] Lin RK, Wu CY, Chang JW, Juan LJ, Hsu HS, Chen CY, Lu YY, Tang YA, Yang YC, Yang PC, Wang YC (2010). Dysregulation of p53/Sp1 control leads to DNA methyltransferase-1 overexpression in lung cancer. Cancer Res.

[25] Yu J, Peng Y, Wu LC, Xie Z, Deng Y, Hughes T, He S, Mo X, Chiu M, Wang QE, He X, Liu S, Grever MR (2013). Curcumin down-regulates DNA methyltransferase 1 and plays an anti-leukemic role in acute myeloid leukemia. PLoS One.

[26] Marin M, Karis A, Visser P, Grosveld F, Philipsen S (1991). Transcription factor Sp1 is essential early embryonic development but dispensable for cell growth and differentiation. Cell.

[27] Takami Y, Russell MB, Gao C, Mi Z, Guo H, Mantyh CR, Kuo PC (2007). Sp1 regulates osteopontin expression in SW480 human colon adenocarcinoma cells. Surgery.

[28] Filicori M (2015). Clinical roles and applications of progesterone in reproductive medicine: an overview. Acta Obstet Gynecol Scand.

[29] Staun-Ram E, Goldman S, Gabarin D, Shalev E (2004). Expression and importance of matrix metalloproteinase 2 and 9 (MMP-2 and -9) in human trophoblast invasion. Reprod Biol Endocrinol.

[30] Kishikawa S, Murata T, Kimura H, Shiota K, Yokoyama KK (2002). Regulation of transcription of the Dnmt1 gene by Sp1 and Sp3 zinc finger proteins. European Journal of Biochemistry.

[31] Chen JZ, Wong MH, Brennecke SP, Keogh RJ (2011). The effects of human chorionic gonadotrophin, progesterone and oestradiol on trophoblast function. Molecular and Cellular Endocrinology.

[32] Van Dijk M, Oudejans C (2014). (Epi)genetic control of human trophoblast invasion. Front Genet.

[33] Gamble KL, Resuehr D, Johnson CH (2013). Shift work and circadian dysregulation of reproduction. Front Endocrinol (Lausanne).

[34] Pliushch G, Schneider E, Weise D, El Hajj N, Tresch A, Seidmann L, Coerdt W, Müller AM, Zechner U, Haaf T (2010). Extreme methylation values of imprinted genes in human abortions and stillbirths. Am J Pathol.

[35] Hanna CW, McFadden DE, Robinson WP (2013). DNA Methylation Profiling of Placental Villi from Karyotypically Normal Miscarriage and Recurrent Miscarriage. The American Journal of Pathology.

[36] Chen Y, Wang K, Leach R (2013). 5-Aza-dC treatment induces mesenchymal-to-epithelial transition in 1st trimester trophoblast cell line HTR8/SVneo. Biochem Biophys Res Commun.

[37] Logan PC, Mitchell MD, Lobie PE (2013). DNA methyltransferases and TETs in the regulation of differentiation and invasiveness of extra-villous trophoblasts. Front Genet.

[38] Zhao C, Meng A (2005). Sp1-like transcription factors are regulators of embryonic development in vertebrates. Development Growth & Differentiation.

[39] Thomas K, Wu J, Sung DY, Thompson W, Powell M, McCarrey J, Gibbs R, Walker W (2007). SP1 transcription factors in male germ cell development and differentiation. Molecular and Cellular Endocrinology.

[40] Norwitz ER, Schust DJ, Fisher SJ (2001). Implantation and the survival of early pregnancy. N Engl J Med.

[41] Moriyama I, Sugawa T (1972). Progesterone facilitates implantation of xenogenic cultured cells in hamster uterus. Nat New Biol.

[42] Jo YS, Lee GS, Nam SY, Kim SJ (2015). Progesterone Inhibits Leptin-Induced Invasiveness of BeWo Cells. International Journal of Medical Sciences.

[43] Goldman S, Shalev E (2006). Difference in Progesterone-Receptor Isoforms Ratio Between Early and Late First-Trimester Human Trophoblast Is Associated with Differential Cell Invasion and Matrix Metalloproteinase 2 Expression. Biol Reprod.

[44] Rubel CA, Lanz RB, Kommagani R, Franco HL, Lydon JP, DeMayo FJ (2012). Research resource: Genome-wide profiling of progesterone receptor binding in the mouse uterus. Mol Endocrinol.

