# Supplementary material for: BMAL1 facilitates trophoblast migration and invasion via SP1-DNMT1/DAB2IP pathway in recurrent spontaneous abortion
Source: Oncotarget. 2017 Sep 7;8(52):89451–64. doi: 10.18632/oncotarget.20702 (PMC5685683; doi:10.18632/oncotarget.20702)
Supplement: Supplementary file 1 [file oncotarget-08-89451-s001.pdf]

BMAL1 facilitates trophoblast migration and invasion via SP1-DNMT1/DAB2IP pathway in recurrent spontaneous abortion

SUPPLEMENTARY MATERIALS

Supplementary Table 1. Clinical information of patients

Subject	Induced Abortion (IA, mean ± S.D., n = 50)	Sporadic Abortion (SA, mean ± S.D., n = 38)	Recurrent Spontaneous Abortion (RSA, mean ± S.D., n = 11)
Age (years)	26.62 ± 5.61	28.10 ± 5.22	29.55 ± 4.44
Gestation age (weeks)	7.92 ± 2.09	8.83 ± 2.26	8.54 ± 2.91
Number of abortions	0.36 ± 0.64	1.45 ± 0.50	3.91 ± 0.79
Parental chromosomes	Not detected	No identification of chromosomal abnormalities	No identification of chromosomal abnormalities
Fetal chromosome	Not detected	Not detected	No identification of chromosomal abnormalities

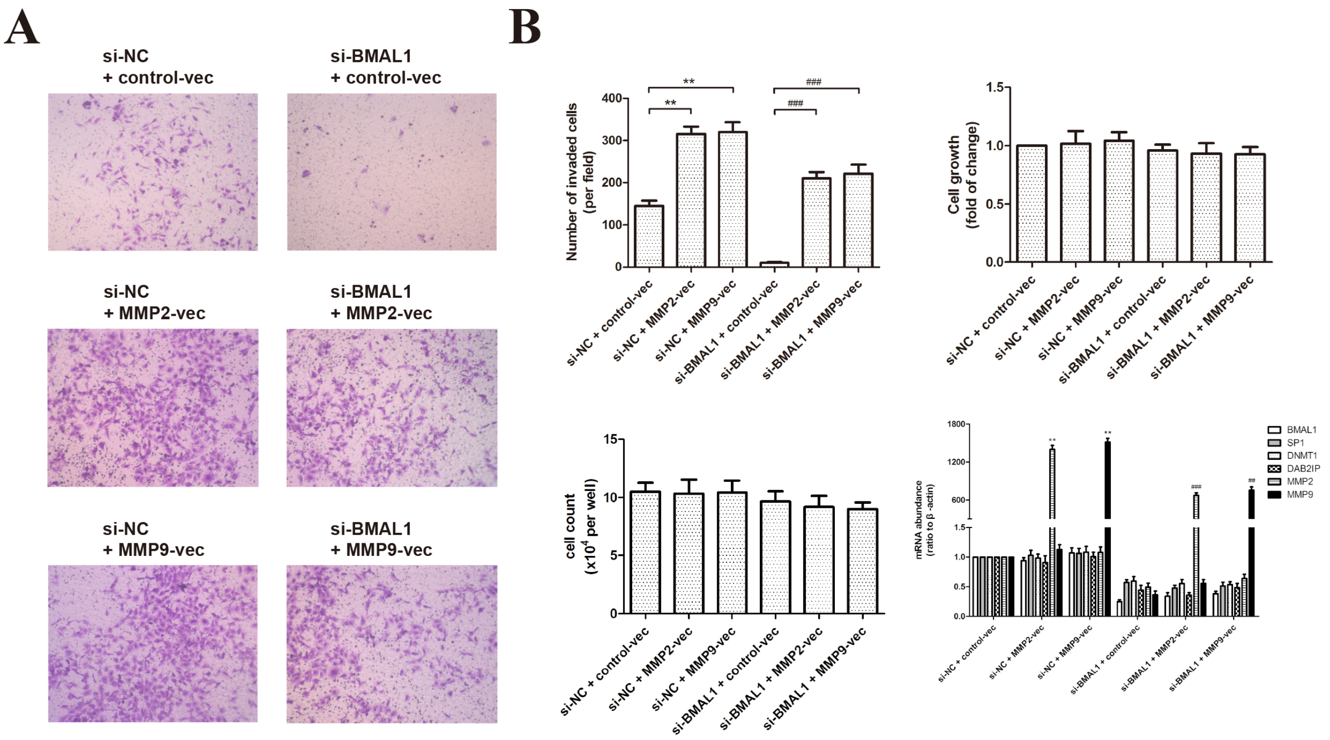

Supplementary Figure 1: BMAL1 induced migration and invasion of HTR-8/SVneo cells via MMP2 and MMP9. (A) Representative images of transwell assay after *BMAL1* knock-down and further *MMP2* or *MMP9* over-expression (magnification: 100 ×). (B) The top panel from left to right is the statistic result of invaded cells and the statistic result of MTT. The bottom panel from left to right is the cell count and the mRNA abundance of *BMAL1, SP1, DNMT1, DAB2IP, MMP2* and *MMP9* after *BMAL1* knock-down and further *MMP2* or *MMP9* over-expression. Images are representative, and data are means ± SEM from three experiments. * $P < 0.05$, ** $P < 0.01$, *** $P < 0.001$ against si-NC + control-vec cells; # $P < 0.05$, ## $P < 0.01$, ### $P < 0.001$ against si-BMAL1 + control-vec cells.
